# A Duplex-Droplet Digital PCR Assay for Simultaneous Quantitative Detection of *Monilinia fructicola* and *Monilinia laxa* on Stone Fruits

**DOI:** 10.3389/fmicb.2021.747560

**Published:** 2021-11-29

**Authors:** Celeste Raguseo, Donato Gerin, Stefania Pollastro, Caterina Rotolo, Palma Rosa Rotondo, Francesco Faretra, Rita Milvia De Miccolis Angelini

**Affiliations:** Department of Soil, Plant and Food Sciences, University of Bari, Bari, Italy

**Keywords:** ddPCR, quantitative PCR (qPCR), brown rot, detection, plant pathogens, fungi, stone fruit

## Abstract

Brown rot, caused by different *Monilinia* species, is a most economically important disease of pome and stone fruits worldwide. In Europe and in Italy, the quarantine pathogen *M. fructicola* was recently introduced and rapidly spread and, by competing with the main indigenous species *Monilinia fructigena* and *Monilinia laxa*, caused relevant changes in *Monilinia* populations. As a result, in most areas, the pathogen almost replaced *M. fructigena* and now coexists with *M. laxa*. The availability of specific and easy-of-use quantification methods is essential to study the population dynamics, and in this work, a new method for the simultaneous quantification of *M. fructicola* and *M. laxa* based on droplet digital PCR (ddPCR) technique was established. Under the optimized reaction conditions, consisting of 250/500 nM of primers/probe sets concentration, 58°C as annealing temperature and 50 PCR cycles, the duplex-ddPCR assay was 200-fold more sensitive than duplex-real-time quantitative PCR (qPCR) assay, quantifying < 1 copy μL^–1^ of target DNA in the PCR mixture. The results obtained with the validation assay performed on apricot and peach fruits, artificially inoculated with conidial suspensions containing different ratios of *M. fructicola* and *M. laxa*, showed a high correlation (*R*^2^ = 0.98) between the relative quantity of DNA of the two species quantified by ddPCR and qPCR and a more accurate quantification by ddPCR compared to qPCR at higher concentrations of *M. fructicola*. The herein described method represents a useful tool for the early detection of *Monilinia* spp. on stone fruits and for the improving knowledge on the epidemiology of brow rot and interactions between the two prevalent *Monilinia* species.

## Introduction

*Monilinia fructicola* (G. Winter) Honey and *Monilinia laxa* (Aderh. & Ruhland) Honey are the main fungal pathogens causing brown rot on peach, apricot, and other stone fruit (De Cal et al., 2009), whereas *Monilinia fructigena* (Pers.) Honey is prevalently found on pome fruit (Lane, 2002). The disease may be responsible for severe yield losses in pre- and postharvest, with early infections appearing as blossom and twig blight or cankers and later infections causing rot on ripening fruit in the field and in postharvest.

*Monilinia laxa* is considered indigenous to Europe, while *M. fructicola* was first introduced in 2001 in France (OEPP/EPPO, 2002), reported in 2009 in Northern Italy (OEPP/EPPO, 2009; Pellegrino et al., 2009), and later also in other regions of the country (Abate et al., 2018). It is currently included in the A2 list of pathogens recommended for regulation as quarantine pests in the EPPO areas.^1^

Monitoring programs carried out in Mediterranean countries, such as Spain (Villarino et al., 2013), Greece (Papavasileiou et al., 2015), and South of Italy (Abate et al., 2018), over the last decade showed changes in the composition of *Monilinia* populations with *M. fructicola* almost completely displacing the indigenous species *M. fructigena* and coexisting with *M. laxa* (Papavasileiou et al., 2015; Abate et al., 2018). The shift caused by *M. fructicola* introduction in new geographic areas could be explained by its faster growth rate, infection, and adaptation to hot–dry conditions, associated with more abundant sporulation compared with *M. laxa* and *M. fructigena* (Byrde and Willetts, 1977; Lichtemberg et al., 2014; Villarino et al., 2016; Abate et al., 2018).

Different *Monilinia* species cause very similar symptoms on infected plant tissues and the identification based on morphological features is labor and time consuming and requires well-trained personal with mycological skills.

Several molecular tools based on the polymerase chain reaction (PCR) of species-specific genetic markers are available and can assist in quick and accurate identification of *Monilinia* species (Hughes et al., 2000; Ioos and Frey, 2000; Côté et al., 2004; Gell et al., 2007; Van Brouwershaven et al., 2010; Hu et al., 2011).

Both quantitative real-time PCR (qPCR) and droplet-digital PCR (ddPCR) are highly sensitive molecular techniques for the quantitative determination of target DNA sequences and represent a significant advancement with respect to conventional PCR (Hindson et al., 2013). In particular, qPCR is widely used for the detection and quantification of plant pathogenic fungi (Schena et al., 2004; Atallah et al., 2007). It allows the quantification of target DNA by monitoring the progression of the reaction using a variety of fluorescent reporter chemistries, also in the same reaction, thus allowing to analyze more than one target (multiplex assays) (Rahman et al., 2013). Quantitative data are obtained by comparing the quantification cycle (Cq) of each sample with those of reference samples containing known DNA amounts to generate a calibration curve (Hindson et al., 2011). qPCR-based methods have been developed for rapid and sensitive detection and quantification of single or multiple *Monilinia* species (Van Brouwershaven et al., 2010; Wang et al., 2018; Ortega et al., 2019). In our previous work, a TaqMan duplex-qPCR assay using species-specific primers/probe sets was developed for simultaneous detection and quantification both of *M. fructicola* and *M. laxa* (Abate et al., 2018). ddPCR, on the other hand, is considered an emerging and efficient molecular technology that provides an alternative, highly sensitive method for absolute quantification of nucleic acids, even with very low abundant targets, being not dependent from calibration (Hindson et al., 2011; Pinheiro et al., 2012). In brief, the ddPCR mixture is partitioned into thousands of water-in-oil droplets, containing zero, one, or more copies of the target nucleic acid, assorted in a random fashion, and each droplet represents an independent nano-PCR event (Yang et al., 2014). After PCR amplification, at the endpoint, the fluorescence of each droplet is individually measured and defined as positive (presence of PCR product) or negative (absence of PCR product). The absolute number of target DNA copies in a sample can then be calculated directly from the ratio of positive events to total partitions using binomial Poisson statistics (Pinheiro et al., 2012).

A wide range of research and diagnostic sectors can benefit from ddPCR applications (Huggett et al., 2013; Gutiérrez-Aguirre et al., 2015; Morcia et al., 2020). ddPCR assays achieved higher accuracy and precision than preexisting methods for the diagnosis of cancer (Olmedillas-López et al., 2017), environmental analyses, controls of food safety, detection of genetically modified organisms (BogožalecKošir et al., 2019), expression analysis (Taylor et al., 2017), and detection and quantification of human and animal parasites and pathogens (Li et al., 2018). Recently, ddPCR has been used for the diagnosis of various plant pathogens such as viruses (Zhang et al., 2017; Liu et al., 2019), phytoplasma (Bahar et al., 2018), and bacteria (Dreo et al., 2014; Dupas et al., 2019). For phytopathogenic fungi, ddPCR has been used to quantify population dynamics of *Aspergillus* species, including major mycotoxin-producing fungi (Palumbo et al., 2016), and recently for the measurement of fungal abundance in soil and plant tissues of *Ilyonectria liriodendri*, associated with black foot disease (del Pilar Martínez-Diz et al., 2020); *Cadophora luteo*-*olivacea*, associated with grapevine Petri disease and esca (Maldonado-González et al., 2020); and *Tilletia* spp., responsible for bunt of wheat (Liu et al., 2020; Xu et al., 2020). Compared to conventional and qPCR, ddPCR assays were reported to be more sensitive and less prone to negative interference by PCR inhibitors (e.g., polyphenols, polysaccharides, and pectin) contained in fruits and other plant parts, food, soil, or water, which can lead to false negative results or an underestimation of the pathogen abundance in the analyzed samples (Zhao et al., 2016; Dupas et al., 2019).

A deeper understanding of brown rot epidemiology and multiple interactions among *M. fructicola*, *M. laxa*, their host plants, and the environment may generate important benefits to the disease management. The aim of the present work was to develop a duplex-ddPCR assay to improve simultaneous and quantitative detection of *M. fructicola* and *M. laxa* in peach (*Prunus persica*) and apricot (*Prunus armeniaca*) fruits. The qPCR assay developed by Abate et al. (2018) was adapted and optimized to ddPCR format, and the performance of the two methods was comparatively assessed in terms of sensitivity, specificity, and replicability. The qPCR and ddPCR assays were then applied to determine relative abundance of the two *Monilinia* species, at different time points, in peach and apricot fruits artificially inoculated with one or both pathogens at different ratios.

## Materials and Methods

### Fungal Isolates and Growing Condition

*Monilinia fructicola* strain Mfrc123 (CBS 144850) and *M. laxa* strain Mlax297 (CBS 144852) used in this work were originally isolated from the fruit of *Prunus avium* collected from commercial orchards in South Italy (Apulia region). Both strains were stored in aqueous 10% glycerol at −80°C and revitalized on potato dextrose agar medium (PDA: infusion from 200 g peeled and sliced potatoes kept at 60°C for 1 h, 20 g dextrose, adjusted at pH 6.5, and 20 g agar Oxoid No. 3 per liter) to obtain fresh cultures. For conidia production, they were grown on PDA supplemented with 25% of commercial vegetable juice (V8^®^, Campbell Soup Company, Camden, NJ, United States) (PDA-V8) and incubated at 25°C ± 1°C in the darkness for the first 3 days and then exposed to a combination of two daylight (Osram, L36W/640) and two near-UV (Osram, L36/73) lamps with a 12 h light/dark photoperiod.

### Fruit Inoculation

Healthy fruits of peach cv. Andros and apricot cv. Pellecchiella, with no visual defects were collected from Apulian orchards conducted according to organic farming. The stage of maturity was verified on 10 apricot and peach fruits, representative of the samples, by using a digital refractometer (Hanna Instruments, Italy) to determine the Brix degrees (peach, 11.5 ± 1.1; apricot, 17.2 ± 1.0) and, only for peaches, a digital penetrometer (8 mm tip, FM200, PCE Italia srl, Italy) to measure the flesh firmness (2.9 ± 0.5 kg/0.5 cm^2^).

Conidia suspensions of *M. fructicola* and *M. laxa* obtained in water added with 0.01% of Tween 20 (Sigma-Aldrich, St. Louis, MO, United States) from 10-day-old cultures were filtered through a layer of Miracloth (Calbiochem, San Diego, CA, United States) to remove mycelium fragments and adjusted to 1–2 × 10^6^ conidia ml^–1^ by using a hemocytometer. The vitality of conidia was assessed, evaluating the conidia germination on PDA disks after 24 h of incubation at 24°C ± 1°C in darkness.

All fruits were decontaminated by immersion in 2% sodium hypochlorite for 3 min, washed twice with sterilized distilled water, and air dried at room temperature before use.

To evaluate the within-host competition between the two *Monilinia* species, fruits were wounded by a sterile needle (5 mm deep) and inoculated with mixtures of conidia suspensions in different *M. fructicola*/*M. laxa* ratios (100:0, 90:10, 50:50, 10:90, and 0:100). Aliquots of 25 μL (apricot, 2 × 10^6^ conidia ml^–1^) or 50 μL (peach, 10^6^ conidia ml^–1^) of each mixture were spotted onto four and six inoculation points of each wounded apricot and peach fruit, respectively. Fruits were then placed on decontaminated Nestipacks (Nespak, Massa Lombarda, Italy) and incubated in a moist chamber at 25°C ± 1°C in darkness. Three fruits for each of three independent replications (in total nine replicate fruits) were used for each combination of fruit and conidia ratios. Fruits inoculated with sterile distilled water with 0.01% of Tween 20 were used as negative control. At 24, 48, and 72 h postinoculation (hpi), the diameters (mm) of rotted areas were recorded. For PCR analyses, at each time point, the areas around the inoculation points were collected from all fruits of each replication with the help of a sterile corkborer (5 mm deep and 10 mm diameter), put together in a tube, immediately frozen with liquid nitrogen, and stored at −80°C until further processing.

### DNA Extraction

Genomic DNA was extracted from 3-day-old mycelium from pure cultures of the two *Monilinia* strains grown at 25°C ± 1°C on cellophane disks overlaid on PDA according to De Miccolis Angelini et al. (2010). For apricot and peach fruits, a modified cetyltrimethyl ammonium bromide (CTAB) protocol for rapid DNA extraction from plant tissues (Li et al., 2008) was used. Briefly, about 2.5 g of fruit was placed into extraction bags (Bioreba, Reinach, Switzerland) with a plastic intermediate layer, homogenized in 10 ml of CTAB extraction buffer [100 mM Tris–Cl, pH 8.0; 1.4 M NaCl; 20 mM ethylenediaminetetraacetic acid (EDTA), pH 8.0; 2% cetyltrimethylammonium bromide (w/v)] using the semiautomated Homex 6 apparatus (Bioreba). An aliquot of 1 ml of each homogenized sample was then transferred into a new tube and incubated at 65°C ± 1°C for 30 min. After extraction with 1 vol of cold chloroform/isoamyl alcohol (24:1, v/v) solution, clear supernatant was precipitated with 0.7 vol of isopropanol at −20°C per 30 min. The tube was then centrifuged at 14,000 rpm for 25 min at 4°C, and the pellet was washed with 500 μL of cold 70% ethanol, air-dried, and dissolved in 50 μL of ultrapure water. DNA quality and concentration were assessed using NanoDrop 2000 spectrophotometer (Thermo Fischer Scientific Inc., Wilmington, DE) and Qubit 2.0 fluorometer (Life Technologies Ltd., Paisley, United Kingdom) with dsDNA BR Assay kit (Thermo Fisher Scientific Inc., Wilmington, DE, United States).

### Optimization of the Droplet Digital PCR Assay and Comparison With the Quantitative PCR Assay

The same primers/TaqMan probe sets targeting *M. fructicola* and *M. laxa* were used in both qPCR and ddPCR assays (Table 1; Abate et al., 2018). All primers and probes were custom synthesized and high-performance liquid chromatography (HPLC) purified by external service (Macrogen, Seoul, South Korea).

**TABLE 1:** Primers/TaqMan probe sets (Abate et al., 2018).

Species	Primer/Probe	Sequences (5′–3′)	Amplicon size (bp)
*M. fructicola*	Mfrc-Fw	GAATGTCGTGAAAGGATAATGGAA	79
	Mfrc-Rev	GCTCTTCTCTCCCCTTTCTTTACC	
	Mfrc-Probe	FAM-TACTAGAGAGGTCTACGGGTG- BHQ1	
*M. laxa*	Mlax-Fw	GCCAAGGGCTCCGTAGGTA	65
	Mlax-Rev	CCTTCACGATCTGCCCCTAGT	
	Mlax-Probe	HEX-CGGCAATAGGCACTACG-BHQ1	

ddPCR was performed on QX200 Droplet Digital PCR System (Bio-Rad, Hercules, CA, United States), according to the manufacturer’s instructions. To optimize the ddPCR conditions, two primers/probe concentrations (240/160 nM and 500/250 nM), a thermal gradient with annealing temperature ranging from 56 to 62°C, and different numbers of PCR cycles were tested on samples containing 25, 2.5, and 0.25 ng of *M. fructicola* and *M. laxa* DNA, in simplex or duplex assays.

ddPCR reaction mixture contained 1 × ddPCR™ Supermix for probes (No dUTP) (Bio-Rad), primers and probes labeled with 6′FAM/BHQ-1 (*M. fructicola*) or 6′HEX/BHQ-1 (*M. laxa*), 2 μL of DNA template, and ultrapure water up to 22 μL. A volume of 20 μL of the total mixture was used to generate droplets with the automated Droplet Generator in an eight-channel DG8 cartridge and cartridge holder with 70 μL of Droplet Generation Oil for probes (Bio-Rad). A volume of 40 μL of the generated emulsion was carefully transferred into a 96-well PCR plate (Bio-Rad), heat sealed with pierceable foil using a PX1™ PCR plate sealer (Bio-Rad), and amplified in a T100™ Thermal Cycler (Bio-Rad). PCR amplification was performed with the following cycling parameters: initial denaturation at 95°C for 10 min, followed by 40 or 50 cycles (at a temperature rate of 2°C s^–1^) of denaturation at 94°C for 30 s and a combined annealing/extension step at 56–62°C for 1 min, and a final step at 98°C for 10 min, ending at 4°C. After amplification, the plate was directly transferred to the Droplet Reader, and the QuantaSoft™ software (version 1.7.4, Bio-Rad) was used for data acquisition and data analysis. For each experiment, a fluorescence amplitude threshold line was manually set up to discriminate positive droplets, with higher fluorescent signals, and negative droplets, with lower fluorescent signals, the latter considered as background, in accordance with negative controls. ddPCR reactions with fewer than 10,000 generated droplets were excluded from the analysis, and a reaction was considered positive if more than two positive droplets were counted. Poisson statistics was used to calculate the absolute copy number concentration of target DNAs in each sample. All the experiments were carried out in triplicate.

The specificity of the ddPCR assay was verified testing DNA from a panel of fungal species commonly associated to stone fruits, i.e., *Alternaria* sp., *Aspergillus niger*, *Botrytis cinerea*, *Cladosporium* sp., *Colletotrichum* sp., *Fusarium* sp., *Monilinia fructigena*, *Monilinia polystroma*, *Mucor* sp., *Penicillium rubens*, *Penicillium expansum*, *Phomopsis amygdali*, *Sclerotinia sclerotiorum*, *Trichoderma* sp., and *Wilsonomyces carpophilus*. The analytical sensitivity for both *M*. *fructicola* and *M*. *laxa* was assessed testing 10-fold serial dilutions of fungal DNA (from 25 to 0.05 pg). The linear regression of the ddPCR assay was determined by plotting the log10 of known DNA amounts against log10 of the number of DNA copies μL^–1^ quantified. Negative controls (NC, healthy peach, or apricot fruit DNAs) and no template control (NTC, ultrapure water) were always included in the experiments. To evaluate the reproducibility of the ddPCR assays, triplicate experiments were performed as independent replicates using DNA samples from fruit spiked with serial dilutions of fungal DNA (25, 2.5, and 0.25 ng) of *M*. *fructicola* and *M*. *laxa*.

ddPCR was compared with qPCR. The qPCR amplifications were performed in a CFX96™ Real-Time PCR Detection System Thermal Cycler (Bio-Rad), according to Abate et al. (2018). The amplification mixture consisted of 1 × Sso Advanced™ Universal Probes Supermix (Bio-Rad), 240 nM of each primer and 160 nM of each probe, 2 μL of DNA template, and ultrapure water up to 12.5 μL. Cycling conditions were 95°C for 3 min, followed by 30 cycles of 95°C for 10 s and 64°C for 30 s. Calibration curves were constructed for 10-fold serial dilutions of DNA (from 25 to 0.05 ng) of *M*. *fructicola* and *M. laxa*. Appropriate controls (i.e., NC and NTC) were included in each run. Every sample was measured in replicates. The Cq values, efficiency (E) of the reaction, coefficient of determination (R^2^), and slope were calculated using the CFX Manager™ software (version 3.1, Bio-Rad Laboratories, RRID: SCR_017251).

The performances of optimize ddPCR and qPCR assays in detecting *M*. *fructicola* and *M*. *laxa* in fruit samples were compared. In detail, the same DNA extracts from fruits artificially inoculated with different ratios of *M. fructicola* and *M. laxa* conidia were used as templates in the two PCR assays, and fungal quantities measured as amount (qPCR) or copy numbers (ddPCR) of each target DNA (*M*. *fructicola* and *M*. *laxa*) were used to estimate the percent proportion of the two fungi in the analyzed sample. For both ddPCR and qPCR, each sample was amplified in duplicate in the same PCR run.

### Statistical Analysis

The data of fruit lesion diameters were analyzed by ANOVA followed by Tukey’s honestly significant different (HSD) test at the significance levels *p* ≤ 0.05 and *p* ≤ 0.01, using CoStat-software (CoHort Software, Monterey, CA, United States). Repeatability and reproducibility of ddPCR and qPCR were evaluated by measuring the intra- and interassay coefficients of variation (CVs).

The ratios of *M*. *fructicola* and *M*. *laxa* quantified by ddPCR were compared with those obtained by qPCR by using χ^2^-test. To compare the two PCR assays, Pearson correlation, linear regression, and the related probability value for each fungal species (*M*. *fructicola* and *M*. *laxa*), fruits (apricot and peach), and sampling times (24, 48, and 72 hpi) were calculated in GraphPad version 6.01 software (La Jolla, CA, United States, RRID: SCR_000306).

## Results

### Optimization of the Duplex-Droplet Digital PCR Assay

**FIGURE 1:**
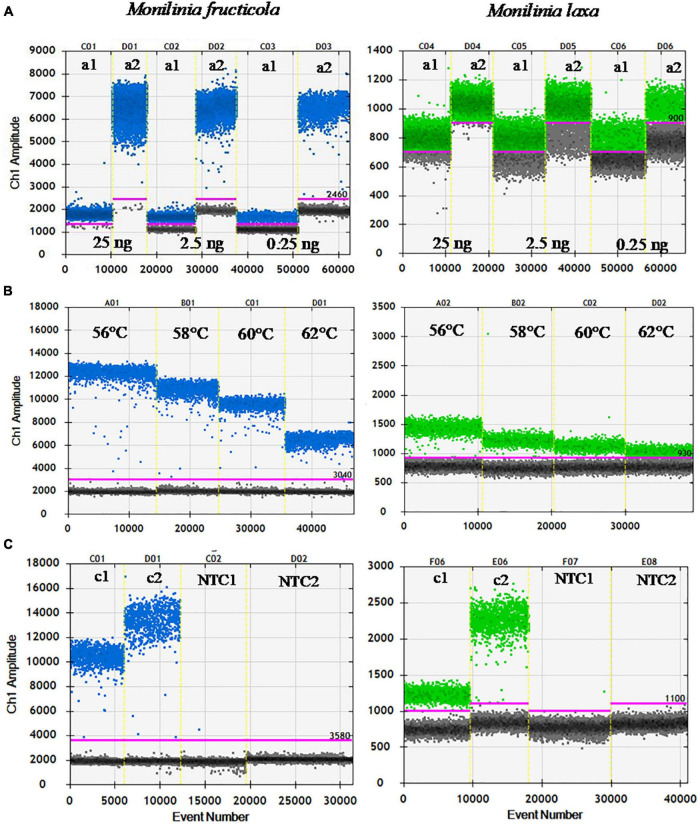
Optimization of ddPCR parameters. **(A)** Primers/probe concentration [240/160 nM (a1) and 500/250 nM (a2)], using 25, 2.5, and 0.25 ng of *M. fructicola* (Mfrc) and *M. laxa* (Mlax) DNAs, at 62°C of annealing temperature, and 40 PCR cycles. **(B)** Annealing temperatures (56, 58, 60, and 62°C) using 2.5 ng of Mfrc and Mlax DNA, 500/250 nM of primers/probe concentration, and 40 PCR cycles. **(C)** PCR cycles number [40 (c1) and 50 (c2)] using 2.5 ng of target DNA, 500/250 nM of primers/probe concentration and 58°C as annealing temperature. NTC, no template control. Blue and green dots represent the positive droplets, above the pink horizontal threshold, for ddPCR with FAM (Mfrc) and HEX (Mlax) probes, respectively. Gray dots represent the negative droplets.

The ddPCR assay was initially set up to identify the optimal primers and probe concentrations, annealing temperature, and cycle number. Figure 1 shows the one-dimensional plots of fluorescence amplitudes at FAM channel, used for *M. fructicola*-specific detection, and at the HEX channel, used for *M. laxa*-specific detection. Droplet separation in the FAM channel was visibly better than in the HEX channel. Almost complete saturation of positive droplets was always obtained at 25 ng of target DNA. For both the primers/probe sets, increased concentrations up to 500/250 nM compared to those used in qPCR (240/160 nM) resulted in higher fluorescence amplitude and better separation between positive and negative droplets, which was clearly visible for *M. fructicola* but not *M. laxa* (Figure 1A). A further increase in concentration of primers up to 900 nM, as recommended by suppliers (Droplet Digital™ PCR Applications Guide), did not improve the droplet patterns in the ddPCR assay and DNA quantification (Supplementary Table 1). To determine the optimal annealing temperature in separating positive and negative droplets, four different annealing temperatures in thermal gradient, including 56, 58, 60, and 62°C, were compared. The best droplet separation for both targets was observed at the lowest tested temperatures, and therefore, 58°C was identified as the most suitable annealing temperature, since it presented slightly less droplet rain between positive and negative droplets compared to 56°C (Figure 1B). Different numbers of PCR cycles were also tested to improve cluster separation for *M. laxa* quantification. An increase in cycle numbers up to 50 cycles yielded higher fluorescence values than 40 cycles and always largest differences in fluorescence signals between positive and negative droplets without inducing false positive results, since no positive droplets were observed for negative controls (NC and NTC) (Figure 1C). Similar results were obtained using different amounts of DNA targets (2.5 and 0.25 ng).

Therefore, the best results for ddPCR were obtained by the combination of 500/250 nM primers/probe concentration, an annealing temperature of 58°C, and 50 PCR cycles. These conditions were used in subsequent ddPCR experiments.

To exclude cross-reactions among primers, probe sets, and the targeted DNAs in duplex ddPCR assay, the optimized protocol was validated using templates containing *M. fructicola* and *M. laxa* DNAs (25–0.25 ng), in both singleplex and duplex formats, with no significant differences in cluster patterns and in the number of DNA copies detected (data not shown). Detection and quantification of fungal DNAs were successful and produced similar results when 2 μL of DNA extract from either peach or apricot healthy fruit was added to the reaction mixture (Supplementary Table 1).

### Performance of the Duplex-Droplet Digital PCR Assay and Comparison With Quantitative PCR Assays

As to duplex-ddPCR specificity, only samples containing DNA from *M. fructicola* and/or *M. laxa* proved positive, while no positive fluorescence signals were detected when using DNAs from non-target *Monilinia* species (e.g., *M*. *fructigena* and *M*. *polystroma*) and other pathogenic and non-pathogenic microorganisms commonly associated with stone fruits.

**FIGURE 2:**
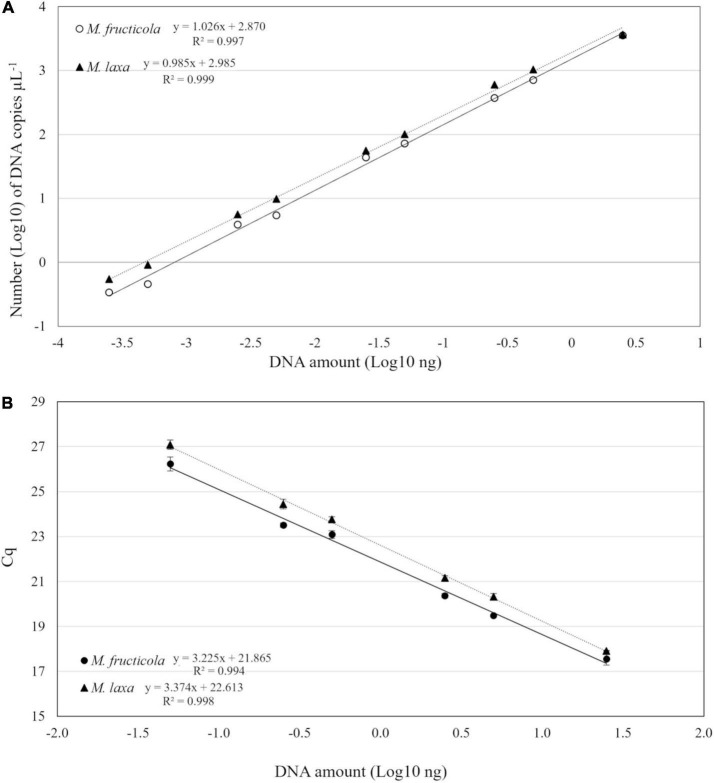
Linear regression of droplet digital PCR (ddPCR) copy numbers **(A)** and real-time PCR assay Cq values **(B)** vs. genomic DNA of *Monilinia fructicola* and *Monilinia laxa*.

The performance of duplex-ddPCR was compared with qPCR for analytical sensitivity, linearity, and dynamic range. Quantitative linearity of both methods was assessed by quantification of serial dilutions (25 ng–0.05 pg) of *M. fructicola* and *M. laxa* DNAs. The sensitivity of detection of qPCR was 0.05 ng for both fungal species. In ddPCR, the lowest detectable DNA amount with reliable positive results for both targets was 0.25 pg, corresponding to 0.34 copies μL^–1^ for *M. fructicola* and 0.44 copies μL^–1^ for *M. laxa* in the PCR mix. Thus, compared to qPCR, ddPCR increased analytical sensitivity by 200-fold. A linear regression model was used to compare for both *Monilinia* species the log10-transformed copy numbers (c) of DNA measured by ddPCR against the corresponding log10-transformed DNA amounts (Figure 2A). For both pathogens, droplets were positively saturated in samples containing 25 ng of target DNA, making the Poisson algorithm invalid and causing a loss of linearity, while the dynamic range of quantification was from 2.5 ng to 0.25 pg with R^2^ values of 0.997 and 0.999 (*p* < 0.0001) for *M. fructicola* and *M. laxa*, respectively. The qPCR assay exhibited a good linearity (*R*^2^ = 0.994 for *M. fructicola* and *R*^2^ = 0.998 for *M. laxa*, *p* < 0.0001) over the dynamic range from 25 to 0.05 ng. The slopes were −3.22 and −3.37, equivalent to an average PCR efficiency of 104.2 and 99.9% for *M. fructicola* and *M. laxa*, respectively (Figure 2B).

To evaluate ddPCR repeatability and reproducibility, templates containing different amounts of fungal target DNA were tested in triplicate in one run and in three independent experiments. The intra-assay CV was included between 0.01 and 0.10, for target DNA templates of both targets ranging from 0.5 ng to 25 pg, while CV values increased (0.14–0.21) when extreme, highest (2.5 ng) or lowest (5 pg) DNA quantities were used. The interassay CV ranged between 0.06 and 0.09 for *M. fructicola* and between 0.07 and 0.11 for *M. laxa*.

### Quantification of *M. fructicola* and *M. laxa* in Artificially Inoculated Apricot and Peach Fruits

**FIGURE 3:**
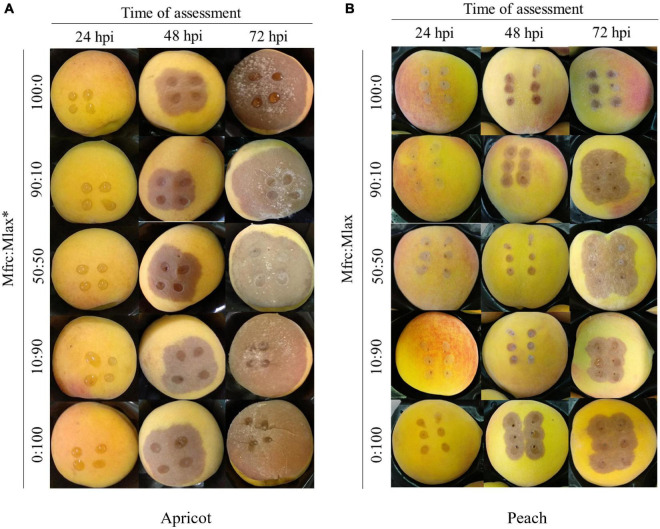
Apricot **(A)** and peach **(B)** fruits artificially inoculated with different *Monilinia fructicola* (Mfrc) and *M. laxa* (Mlax) conidial suspension ratios (*).

At 8 hpi, the percentages of germinated conidia on PDA were 15% (*M. fructicola*) and 20% (*M. laxa*) with the length of the germ tube of 3.42 ± 0.16 μm (*M. fructicola*) and 4.14 ± 0.25 μm (*M. laxa*). At 24 hpi, all conidia germinated on PDA disks without differences among the two species. At that time, no rotting areas were observed on inoculated apricot and peach fruits. In the subsequent assessments, lesions expanded faster on apricot compared to peach fruits, likely due to higher degree of fruit ripeness in apricot compared to peach. In details, at 48 hpi, the lesion ranged from 9.8 to 15.3 mm in diameter on apricot fruits and from 5.3 to 14.2 mm on peach fruits; at 72 hpi, it ranged from 42.2 to 44.6 mm on apricot and from 9.8 to 20.8 mm on peach fruits. At 48 hpi, fruits of apricot and peach inoculated with *M. fructicola* alone or coinoculated with *M. laxa* in the Mfrc/Mlax ratios of 90:10 and 50:50 exhibited a diameter of lesions statistically (*p* ≤ 0.01) smaller than fruits inoculated with *M. laxa* alone or with a large abundance of *M. laxa* (Mfrc/Mlax 10:90). At 72 hpi, the diameter of lesions on peach fruits inoculated with *M. fructicola* were statistically (*p* ≤ 0.01) lower than that caused by *M. laxa* inoculated alone or coinoculated in different ratios with *M. fructicola*, while no statistical difference was observed on apricot (Table 2 and Figure 3).

**TABLE 2:** Lesion diameters on apricot and peach fruits artificially inoculated with different *M. fructicola* and *M. laxa* conidial suspension ratios.

Assessment	(Mfrc/Mlax)*	Lesion diameters (mm)	
		
		Apricot	Peach
24 hpi**	100:0	0.0 ± 0.0	0.0 ± 0.0
	90:10	0.0 ± 0.0	0.0 ± 0.0
	50:50	0.0 ± 0.0	0.0 ± 0.0
	10:90	0.0 ± 0.0	0.0 ± 0.0
	0:100	0.0 ± 0.0	0.0 ± 0.0
48 hpi	100:0	9.8 ± 2.0 c C	6.3 ± 0.4 c C
	90:10	10.5 ± 1.7 bc BC	5.3 ± 0.6 c C
	50:50	13.3 ± 1.3 ab ABC	5.7 ± 0.4 c C
	10:90	13.7 ± 1.7 a AB	9.1 ± 0.8 b B
	0:100	15.3 ± 0.3 a A	14.2 ± 1.2 a A
72 hpi	100:0	42.3 ± 3.5 a A	9.8 ± 1.0 d C
	90:10	43.1 ± 2.9 a A	18.6 ± 2.3 ab AB
	50:50	42.2 ± 3.9 a A	20.8 ± 0.9 a A
	10:90	42.8 ± 2.4 a A	15.6 ± 1.3 c B
	0:100	44.6 ± 2.2 a A	17.0 ± 1.2 bc B

Figures are mean values of nine replicates ± standard error. Data referred to each fruit and sampling time followed by different letters are statistically different at the probability values p = 0.05 (lowercase) or p = 0.01 (uppercase) according to the Tukey’s test. *Ratio of Monilia fructicola (Mfrc) to Monilinia laxa (Mlax) in the conidial inoculum. **hpi, hours postinoculation.

At each time points, DNA extracted from fruit infected areas were analyzed using both ddPCR and qPCR, and the quantitative results obtained by the two methods were compared (Table 3). *M. fructicola* and *M. laxa* were detectable by both the assays in all fruit samples inoculated with each of the two *Monilinia* species alone or together at various ratios and different time points corresponding to different levels of fruit infection and colonization. When *M. fructicola* or *M. laxa* were inoculated alone (Mfrc/Mlax 100:0 or 0:100), no positive reaction with the no-target species was confirmed, strengthening the species specificity of the assays.

**TABLE 3:** Comparison among quantity of *Monilinia fructicola* and *M. laxa* DNAs in duplex-qPCR (ng) and duplex-ddPCR (copies μL^–1^) assays on artificially inoculated apricot and peach fruits.

Mfrc/Mlax^a^	*M. fructicola^b^*						*M. laxa*					
		
	24 hpi		48 hpi		72 hpi		24 hpi		48 hpi		72 hpi	
						
	qPCR	ddPCR	qPCR	ddPCR	qPCR	ddPCR	qPCR	ddPCR	qPCR	ddPCR	qPCR	ddPCR

Apricot												
100:0	6.1 ± 0.9	256.0 ± 27.5	8.1 ± 2.7	264.0 ± 58.3	31.7 ± 6.1	94.3 ± 6.4	n.d.	n.d.	n.d.	n.d.	n.d.	n.d.
90:10	5.8 ± 1.0	236.0 ± 27.6	10.1 ± 2.8	324.0 ± 64.3	14.3 ± 3.5	47.8 ± 10.2	0.2 ± 0.0	12.1 ± 0.4	1.3 ± 0.4	76.3 ± 16.4	2.6 ± 0.7	16.6 ± 4.7
50:50	2.0 ± 0.8	88.9 ± 24.7	4.7 ± 0.9	148.0 ± 15.4	8.4 ± 1.2	23.0 ± 0.5	0.5 ± 0.1	43.2 ± 7.0	5.4 ± 1.1	275.3 ± 33.5	7.1 ± 1.5	31.9 ± 3.5
10:90	0.6 ± 0.2	25.3 ± 2.1	0.6 ± 0.1	21.5 ± 1.4	0.3 ± 0.0	1.4 ± 0.4	2.0 ± 0.4	115.0 ± 17.6	8.8 ± 1.3	459.0 ± 45.5	6.3 ± 1.3	34.9 ± 5.3
0:100	n.d.^c^	n.d.	n.d.	n.d.	n.d.	n.d.	3.1 ± 0.8	211.0 ± 38.6	5.4 ± 0.1	325.0 ± 10.4	7.7 ± 0.7	50.7 ± 3.1

r^d^	0.990***		0.978***		0.947***		0.983***		0.982***		0.925***	

**Peach**												

100:0	1.7 ± 0.2	109.2 ± 4.6	8.0 ± 1.7	367.0 ± 63.6	13.2 ± 4.2	49.7 ± 12.1	n.d.	n.d.	n.d.	n.d.	0.2 ± 0.1	1.3 ± 0.7
90:10	2.3 ± 0.5	129.7 ± 24.6	9.1 ± 1.4	394.0 ± 33.3	15.9 ± 1.0	1.6 ± 0.3	0.2 ± 0.0	14.6 ± 3.0	5.5 ± 0.7	450.3 ± 43.1	28.9 ± 1.0	199.0 ± 42.6
50:50	1.0 ± 0.1	53.9 ± 7.4	2.0 ± 0.4	70.4 ± 2.4	4.3 ± 1.1	24.2 ± 5.0	0.4 ± 0.1	44.0 ± 5.8	1.3 ± 0.2	90.7 ± 12.3	47.6 ± 16.9	463.3 ± 163.5
10:90	0.3 ± 0.1	15.7 ± 2.9	0.4 ± 0.1	21.3 ± 2.3	0.5 ± 0.1	61.0 ± 11.6	1.6 ± 0.4	130.3 ± 22.9	1.8 ± 0.2	172.3 ± 18.5	27.9 ± 6.2	215.7 ± 40.9
0:100	n.d.	n.d.	n.d.	n.d.	n.d.	n.d.	1.3 ± 0.1	102.7 ± 16.8	29.5 ± 1.8	318.3 ± 50.3	22.5 ± 4.2	168.0 ± 14.7

r	0.984***		0.992***		0.249		0.984***		0.499		0.819***	

*^a^Ratio of Monilinia fructicola (Mfrc) to M. laxa (Mlax) in conidial inoculum used for artificial inoculation.*

*^b^DNA of M. fructicola and M. laxa quantified by duplex-qPCR and -ddPCR in artificially inoculated apricot and peach fruits. hpi, hours post inoculation.*

*^c^n.d., no detectable (< 0.34 copies μL^–1^ for M. fructicola and < 0.44 copies μL^–1^ for M. laxa for ddPCR, and < 0.05 ng of DNA from both Monilinia species for qPCR).*

^d^Coefficient of correlation (r) obtained from fungal DNA quantified by qPCR and ddPCR based on the Pearson’s correlation at probability value p < 0.005 (***).

The correlation values between the ddPCR and qPCR quantification data for the two pathogens (Table 3) were significant (*p* < 0.005) with *r* ≥ 0.978 at 24 and 48 hpi, and *r* ≥ 0.819 at 72 hpi, with only few exceptions (*M. fructicola* at 72 hpi and *M. laxa* at 48 hpi on peach). The CV mean values among replicates of quantification data were 0.32 (ranging from 0.03 to 0.71) ng for qPCR and 0.26 (ranging from 0.07 to 0.61) copies μL^–1^ for ddPCR.

**FIGURE 4:**
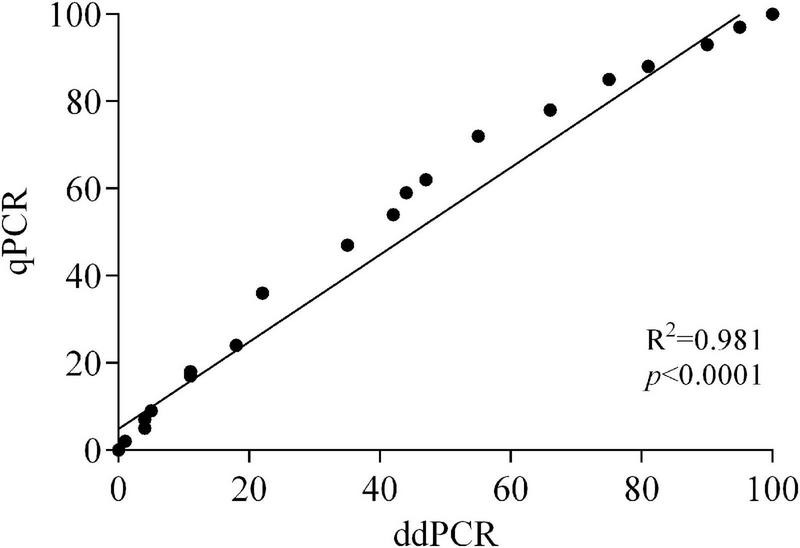
Correlation between ddPCR and qPCR data of *Monilinia fructicola* vs. *Monilinia laxa* ratios in artificially inoculated fruits of apricot and peach.

On both apricot and peach fruit, and at all the sampling times (24, 48, and 72 hpi), linear regression analysis indicated significant accordance between ddPCR and qPCR based on Pearson’s correlation (*R*^2^ = 0.981 and *p* < 0.0001) for the estimation of *M. fructicola* to *M. laxa* DNA ratios in infected fruits (Figure 4).

According to the chi-square test, statistically significant differences (*p* ≤ 0.05) between data of *M. fructicola* to *M. laxa* ratios determined by ddPCR and qPCR were observed at 24 and 48 hpi (50:50 ratio) and at 48 and 72 hpi (90:10 ratio) on both fruits and at 72 hpi (50:50 ratio) only on apricot (Table 4). qPCR slightly overestimated (up to 17%) *M. fructicola* compared to *M. laxa* in all the coinoculated fruit samples, as a possible consequence of the efficiency of *M. fructicola* quantification that was 103.0% (apricot) and 105.4% (peach), while that for *M. laxa* was 100.0% (apricot) and 96.9% (peach). At 24 hpi, the estimated proportions of fungal DNAs showed higher percentages of *M. fructicola* (up to 16%) in the Mfrc/Mlax ratios compared to those in the conidial inoculum used for coinoculations of the two species on both apricot and peach fruits. An increase in the *M. laxa* DNA proportion was generally recorded starting from 48 hpi on both fruits, more markedly on peach (up to 43%) than on apricot (up to 31%). On apricot fruits, the DNA ratios between the two *Monilinia* species at 72 hpi remained more stable, while on peach, a further relative increase in *M. laxa* DNA proportion was observed (up to 39%).

**TABLE 4:** Comparison among ratios of *Monilinia fructicola* (Mfrc) and *M. laxa* (Mlax) DNAs quantified by duplex-ddPCR and duplex-qPCR assays in apricot and peach fruits artificially inoculated with mixed conidial suspensions (1–2 × 10^6^ mL^–1^) containing different ratios of conidia of the two pathogens.

Conidial suspension Mfrc/Mlax^a^	Mfrc/Mlax DNA ratios^b^																	
	
			Apricot							Peach								
					
	24 hpi			48 hpi			72 hpi			24 hpi			48 hpi			72 hpi		
						
	ddPCR	qPCR	χ^2^	ddPCR	qPCR	χ^2^	ddPCR	qPCR	χ^2^	ddPCR	qPCR	χ^2^	ddPCR	qPCR	χ^2^	ddPCR	qPCR	χ^2^
100:0	100:0	100:0	0.000	100:0	100:0	0.000	100:0	100:0	0.000	100:0	100:0	0.000	100:0	100:0	0.000	100:0	100:0	0.000
90:10	95:5	97:3	1.375	97:3	88:12	4.640*	75:25	85:15	7.843**	90:10	93:7	1.382	47:53	62:38	9.550**	22:78	36:64	8.507**
50:50	66:34	78:22	8.392**	78:22	47:53	5.781*	42:58	54:46	5.797*	55:45	72:28	14.335**	44:56	59:41	9.301**	5:95	9:91	1.954
10:90	18:82	24:76	1.974	24:76	7:93	1.382	4:96	4:96	0.000	11:89	17:83	2.551	11:89	18:82	3.320	1:99	2:98	0.510
0:100	0:100	0:100	0.000	0:100	0:100	0.000	0:100	0:100	0.000	0:100	0:100	0.000	0:100	0:100	0.000	0:100	0:100	0.000

Calculated χ^2^-values higher than 3.84 and 6.63 indicate significant differences between data from ddPCR and qPCR at the probability level p = 0.05 (*) and p = 0.01 (**), respectively.

*^a^Ratio of Mfrc to Mlax conidial inoculum used for artificial inoculation.*

*^b^Ratio of Mfrc and Mlax DNAs quantified by duplex-ddPCR and qPCR assays in artificially inoculated apricot and peach fruits. hpi, hours postinoculation.*

## Discussion

The introduction of the quarantine species *M. fructicola* into new areas caused significant changes in *Monilinia* populations responsible for brown rot on stone fruit (Villarino et al., 2013; Abate et al., 2018), so intensive and careful monitoring programs are crucial for evaluating the effectiveness of integrated crop protection strategies and whether adaptation is need (Côté et al., 2004).

The recent availability of ddPCR for the quantification of fungal pathogens allows the absolute quantification of the target, without the use of a standard curve, and an improvement in accuracy and sensitivity as compared with qPCR assays (Palumbo et al., 2016; del Pilar Martínez-Diz et al., 2020). In the present study, a duplex-ddPCR assay for the simultaneous quantification of both *M. fructicola* and *M. laxa*, the main agents of brown rot on stone fruits in Italy and many other Countries, was set up, optimized, and compared to the qPCR assay previously developed by Abate et al. (2018) and successfully applied to quantify the relative proportions of the two species in artificially inoculated apricot and peach fruits. All the experiments meet the minimum requirements for qPCR (Bustin et al., 2009) and digital PCR (Huggett et al., 2013; dMIQE Group and Huggett, 2020) data.

The concentrations of primers/TaqMan probe sets efficiently used in qPCR (240/160 nM), first tested in ddPCR resulted in low amplitude signals of droplets individually analyzed by using a two-color detection system set to detect FAM- and HEX-labeled probes, specific for *M. fructicola* and *M. laxa*, respectively. An increase in the concentrations up to 500/250 nM resulted in a higher fluorescence amplitude for both primers/probe sets and better separation between positive and negative droplets for FAM detection. Moreover, a thermal gradient was used to optimize reaction conditions that allowed to identify 58°C as the best annealing temperature improving droplet separation for both FAM and HEX detection. To further improve the known limited separation between positive and negative droplets with the HEX probe, the number of cycles routinely set up to 40 in ddPCR assays was increased up to 50 cycles as suggested by Huggett et al. (2013) and dMIQE Group and Huggett (2020).

A good and comparable linearity (*R*^2^ ≥ 0.997) in ddPCR vs. qPCR was ascertained for both the fungal species *M. fructicola* and *M. laxa*. In addition, ddPCR was about 200-fold more sensitive than qPCR. The quantification limit using ddPCR was 0.25 pg for both *M. fructicola* and *M. laxa*, while 50 pg of DNA was the quantification limit using qPCR for both pathogens. In details, the detection threshold values of ddPCR on *M. fructicola* and *M. laxa* DNA was of 0.34 and 0.44 copies μL^–1^ in the PCR mix, respectively, for the two species, corresponding to 7.5 and 9.7 copies of the target DNA in the analyzed sample. The results herein reported are consistent with those obtained in other research studies on other plant pathogens (Zhao et al., 2016; Maheshwari et al., 2017; del Pilar Martínez-Diz et al., 2020). Nevertheless, ddPCR failed to quantify more than 4,000 copies μL^–1^ of DNA target corresponding to about 7.5 ng, due to complete saturation of positive droplets at high concentrations of DNA template.

The ddPCR assay showed relatively low intra-/interassay variation, indicating that it can provide a repeatable and reproducible quantification of *M. fructicola* and *M. laxa* in fruits, especially for samples with concentrations of target DNA in the reaction mixture ranging from 25 pg to 0.5 ng corresponding to a number of copies μL^–1^ from 44 (*M. fructicola*) or 56 (*M. laxa*) to 711 (*M. fructicola*) or 1,037 (*M. laxa*).

To validate the duplex-ddPCR assay, apricot and peach fruits were artificially inoculated with different *M. fructicola*/*M. laxa* conidial suspension ratios, and the DNA of both fungal species was quantified by both ddPCR and qPCR and used to estimate the relative *Monilinia* ratios. A high correlation between the two molecular techniques was observed. However, differences in the amplification efficiency of DNA quantification from the two species by duplex qPCR caused an overestimation of *M. fructicola* vs. *M. laxa* and consequently less accurate estimation of their proportion in infected fruits. Indeed, in our qPCR experiments, the average efficiency of the *M. laxa* calibration curve was 98.4%, whereas the efficiency of the *M. fructicola* calibration curve was higher than 100% (104.2%), likely due to interfering compounds in DNA extracts. Therefore, application of ddPCR for detection and quantification of *M. fructicola* and *M. laxa* in stone fruits was more sensitive and reliable. The ddPCR assay is hence a reliable method with enhanced sensitivity and accuracy in detection of the two species even without visible symptoms on fruits and could be reliably applied to analyze latent *Monilinia* infections in plant tissues. No inhibition or loss of sensitivity was indeed observed when *Monilinia* DNA was extracted from peach and apricot fruits.

At all sampling times (24, 48, and 72 hpi) in our experiments, *M. fructicola* and *M. laxa* species were quantified, taking into consideration the initial ratios of the conidial suspension used for the inoculation. A general increase in *M. laxa* DNA was recorded starting from 48 hpi on both apricot and peach fruits, and at 72 hpi on peach became prevalent over *M. fructicola* as compared to the starting ratio. Moreover, the lesions observed on the artificially inoculated fruits with conidial suspensions including *M. laxa* conidia were always higher than those observed on fruits inoculated with *M. fructicola* alone. These results agree with those obtained by Hu et al. (2011) who observed on peach fruits a slower growing of *M. fructicola* compared to *M. laxa*, although they proved a fastest colony growth rate on PDA medium. Thus, the fitness edge proved for *M. fructicola* vs. *M. laxa* in the monitoring programs (Villarino et al., 2013; Papavasileiou et al., 2015; Abate et al., 2018; Ortega et al., 2019) seems to be not strictly related to the pathogenicity, and other competitive factors, such as vegetative vigor and abundance of conidia production, are likely responsible for the replacement of the indigenous species *M. laxa* and *M. fructigena* (Villarino et al., 2013).

A preliminary evaluation of the possible application of the new ddPCR method was carried out on 20 naturally infected fruits of peach, apricot, and cherry from different orchards and three regions of Southern Italy (Apulia, Basilicata, and Campania), and 8 fruits were infected by *M. laxa* and 12 by *M. fructicola* and no mixed infections were found. Currently, the method is applied in more extensive monitoring programs on *Monilinia* populations on stone fruit in different geographic areas.

In conclusion, with this study, a duplex-ddPCR assay for the quantification of *M. fructicola* and *M. laxa* was optimized, validated, and its performance compared with the duplex-qPCR assay. To the best of our knowledge, this is the first study on the application of ddPCR in the quantitative detection of *Monilinia* species. ddPCR could produce highly sensitive and quantitative results. The method could be useful in the future in monitoring programs on *Monilinia* populations, especially at low levels of infection and in the presence of high levels of PCR inhibitors in plants, although the high cost of ddPCR still represents a drawback for its use as a high throughput method. The monitoring of *Monilinia* species present in single orchard or a growing area would support the growers in the implementation of more sustainable and effective crop protection strategies by more appropriate choices of plant protection products against brown rot pathogens during the cropping season, as differences in fungicide responses have been reported for different *Monilinia* species (Egüen et al., 2016; Abate et al., 2018). Based on the results observed through the validation of the assay on artificially inoculated apricot and peach fruits, the methods can be applied to investigate the behavior of the two fungal species and their population dynamics even when they coexist and share the same ecological niche.

## Data Availability Statement

The raw data supporting the conclusions of this article will be made available by the authors, without undue reservation.

## Author Contributions

CRa designed the experiments, performed the main experimental activities, collected and analyzed the data, and wrote the main manuscript. DG performed some experiments, contributed to data analysis, and to the writing of the main manuscript. SP, RD, and CRo designed and performed some experiments, analyzed the data, and wrote the main manuscript. PR analyzed the data. FF contributed to the experiments design, supervised, and complemented the writing and coordinated the collaboration of the authors. All authors reviewed the manuscript.

## Conflict of Interest

The authors declare that the research was conducted in the absence of any commercial or financial relationships that could be construed as a potential conflict of interest.

## Publisher’s Note

All claims expressed in this article are solely those of the authors and do not necessarily represent those of their affiliated organizations, or those of the publisher, the editors and the reviewers. Any product that may be evaluated in this article, or claim that may be made by its manufacturer, is not guaranteed or endorsed by the publisher.

## Footnotes

1
https://www.eppo.int
